# Obsessive-Compulsive and Post Traumatic Avoidance Symptoms Influence the Response to Antihypertensive Therapy: Relevance in Uncontrolled Hypertension

**DOI:** 10.3390/ph2030082

**Published:** 2009-11-16

**Authors:** Anna Realdi, Angela Favaro, Paolo Santonastaso, Marco Nuti, Emanuela Parotto, Giulia Inverso, Matteo Leoni, Luisa Macchini, Francesca Vettore, Lorenzo Calo, Andrea Semplicini

**Affiliations:** 1 Clinica Medica 4, Department of Clinical and Experimental Medicine, Policlinico Universitario, Via Giustiniani 2 35128 Padova, Italy; Emails: emanuela.parotto@unipd.it (E.P.); giulia.inverso@unipd.it (G.I.); matteo.leoni@libero.it (M.L.); luisa.macchini@unipd.it (L.M.); francesca.vettore@unipd.it (F.V.); renzcalo@unipd.it (L.C.); 2 Psychiatric Clinic, Department of Neuroscience, University of Padua, Policlinico Universitario, Via Giustiniani 2 35128 Padova, Italy; Emails: angela.favaro@unipd.it (A.F.); paolo.santonastaso@unipd.it (P.S.); marco.nuti@unipd.it (M.N.); 3 Division of General Medicine, SS. Giovanni e Paolo Hospital, Campo SS. Giovanni e Paolo, Castello 6777–30122 Venezia, Italy; Email: andrea.semplicini@ulss12.ve.it (A.S.)

**Keywords:** avoidance symptoms, compliance, obsessive-compulsive symptoms, uncontrolled hypertension, type D personality

## Abstract

*Aim*: To investigate the association of uncontrolled hypertension with psychological factors associated with high cardiovascular morbidity and mortality (type D personality, depression, posttraumatic stress-related symptoms). *Methods*: 205 consecutive outpatient hypertensives completed three questionnaires evaluating Type D personality (DS 16), post traumatic symptoms (revised Impact of Events Scale), symptoms of anxiety, hostility, depression and obsessive-compulsive traits (subscales of the Symptom Checklist). Uncontrolled hypertension was diagnosed when clinic sitting blood pressure was above 140/90 mmHg (130/80 in the presence of diabetes or nephropathy), despite reported adherence to treatment with at least three antihypertensive medications, including a diuretic. *Results*: Uncontrolled hypertension (39%), was predicted by lower scores at Symptom Checklist obsessive-compulsive subscale and higher number of post traumatic avoidance symptoms, older age, diabetes, higher systolic pressure at first visit and longstanding hypertension. Type D personality correlated with depression, hostility, anxiety, compulsiveness, history of malignancy, and older age, but not with uncontrolled hypertension. *Conclusions*: Uncontrolled hypertension is associated with low obsessionality and avoidance symptoms, which reduce compliance to treatment. On the contrary, type D personality is not correlated with uncontrolled hypertension, as it includes compulsiveness, which improves compliance. A multidisciplinary approach to the hypertensive patient is mandatory to establish if the psychological profile affects compliance.

## Introduction

Resistant hypertension is becoming a clinical entity due to its large prevalence and high associated cardiovascular risk [1,2,3]. It is defined by blood pressure above 140/90 mmHg (130/80 in patients with diabetes or renal disease), despite adherence to an appropriate three drug regimen, including a diuretic.

The inability to lower blood pressure stems from secondary hypertension, lifestyle factors and ingestion of substances that elevate blood pressure. White coat effect, poor compliance, inadequate dose or blood pressure measurement, physician selection of inappropriate drugs all affect blood pressure control and may cause what should be better named as “pseudoresistant” hypertension [1,3]. Recently, Alderman stressed the difficulty of distinguishing “resistant” from “pseudoresistant” hypertension and that, in the absence of rigorous therapeutic criteria of resistant hypertension, the term of “uncontrolled hypertension” should be preferred [4].

Many studies have pointed out the role of psychological factors in hypertension, such as defensiveness, alexithymia, negative affectivity, expression of anger, anxiety and depression [5,6], but very few explored the impact of psychological factors in resistant or uncontrolled hypertension. Anxiety and inability to channel anger were more frequent in resistant hypertension [7,8], but others showed no correlation with psychological variables [9,10]. The aim of our study was, therefore, to evaluate the psychological correlates of uncontrolled hypertension. In particular, we explored the presence of recent stressful and traumatic life events, of posttraumatic stress-related symptoms, the general psychopathology and if ‘type D personality’, which describes patients who experience increased negative emotions and tend to inhibit the expression of emotions in social interactions [11,12,13], has some role in increasing the risk of uncontrolled hypertension.

## Results

In the whole sample (205 patients), 184 (90%) were diagnosed as having essential hypertension and 21 (10%) as secondary hypertension (eight idiopathic hyperaldosteronism due to bilateral adrenal hyperplasia, two aldosterone producing adenoma, four renovascular hypertension, three chronic renal insufficiency, one adult polycystic kidney disease, one aortic coarctation, one Cushing syndrome and one polycythemia vera). The two groups did not differ for clinical and demographic characteristics (age, sex, blood pressure at first and last visit, BMI, number of outpatient consultations, duration of known hypertension, number of antihypertensive drugs, total vascular events, diabetes, malignancy, use of antidepressants or benzodiazepines, data not shown), but the number of cardiovascular events (history of myocardial infarction or angina) was more frequent among the patients with secondary hypertension (3/21 vs. 4/184, χ2 = 8.38; p < 0.005). 

One hundred and twenty six hypertensives were classified as controlled (61.5%), and 79 as uncontrolled (38.5%). Uncontrolled hypertensives were older, had higher baseline systolic blood pressure, longer duration of hypertension, greater number of outpatient consultations, larger prevalence of diabetes and cardiovascular events than responders (Table 1). By definition, uncontrolled hypertensives had also higher blood pressure values at the last visit and were taking a greater number of antihypertensive drugs.

**Table 1:** Demographic and clinical characteristics of “responder” and “resistant” hypertensives.

	Responder (n = 126)	Resistant (n = 79)	p Value
Age (yrs)	53 (±12)	62 (±13)	.000
Male gender	n = 60 (47.6%)	n = 35 (44.3%)	NS
Diagnosis of hypertension			
Essential hypertension	n = 115 (92 %)	n = 69 (86 %)	NS
Secondary hypertension	n = 11 (8%)	n = 10 (14 %)	
BP at first visit			
Systolic BP (mmHg)	155 (±19)	167 (±21)	.001
Diastolic BP (mmHg)	96 (±12)	99 (±14)	NS
BP last visit			
Systolic BP (mmHg)	132 (±10)	153 (±16)	.000
Diastolic BP (mmHg)	82 (±8)	88 (±12)	.001
Outpatient consultations	n = 6 (±5)	n = 10 (±10)	.003
Duration of hypertension (yrs)	8 (±7)	12 (±8)	.000
Antihypertensive drugs	n = 2 (±1)	n = 4 (±1)	.000
BMI	27 (±5)	28 (±4)	NS
Diabetes	n = 10 (8%)	n = 21 (26%)	.000
Vascular diseases	n = 12 (10%)	n = 13 (16%)	NS
Ischemic heart disease	n = 0 (0%)	n = 7 (8%)	.001
History of malignancy	n = 12 (10%)	n = 7 (9%)	NS

NS = not significant; BMI: body mass index (weight (Kg)/ height (m2)); BP = blood pressure values; vascular diseases (angina, myocardial infarct, heart failure, stroke, transitory ischemic accident).

### Type D personality and other psychopathological aspects

One hundred and sixty six patients returned the questionnaires (81%). The clinical and demographic characteristics (age, sex, blood pressure at first and last visit, BMI, number of outpatient consultations, known duration of hypertension, number of antihypertensive drugs, use of antidepressant drugs, type of hypertension, rate of response to treatment, diabetes, malignancy) were similar between the patients who returned the questionnaire and those who did not (data not shown). However, those who returned the questionnaires had fewer total vascular events (16/166 versus 9/39 patients; p= 0.026), history of malignancy (12/166 versus 7/39; p = 0.045) and use of benzodiazepine (14/166 vs. 8/39; p = 0.034). Another 19 subjects were excluded from subsequent analyses because they returned incomplete questionnaires. In the final sample (n = 147), the prevalence of type D personality was 37 % (n = 54). Type D patients were significantly older than non-type D patients (59.6 ± 12.0 yrs vs. 55.2 ± 12.2; p < 0.04), but did not differ for gender, blood pressure at first and last visit, number of consultations, duration of hypertension, number of drugs, BMI, type of hypertension, blood pressure response to antihypertensive treatment, history of diabetes, previous cardiovascular events, use of benzodiazepines or antidepressants. Type D personality was associated with history of malignancy (14% vs. 3%; χ2 = 6.44; p < 0.02; OR = 5.1, 95% C.I. 1.3-20.2).

Type D patients scored significantly higher than non Type D patients at H-SCL obsessive-compulsive (1.3 ± 0.9 vs. 0.7 ± 0.6; p<0.001), depression (1.1 ± 0.6 vs. 0.6 ± 0.6; p<0.001), anxiety (1.1 ± 0.7 vs. 0.7 ± 0.5; p<0.001) and hostility (0.7 ± 0.6 vs. 0.5 ± 0.6; p<0.04) subscales. The presence of recent stressful and traumatic life events and of posttraumatic stress-related symptoms was not different between the two groups.

### Resistance to antihypertensive treatment, type D personality, and psychopathological profile

The psychological characteristics, by response to antihypertensive therapy, are reported in Table 2.

**Table 2:** Psychological characteristics of “responder” and “resistant” hypertensives.

DS-16	Responder(n = 88)	Resistant(n = 59)	p Value
Negative affectivity	12.3 (7.0)	12.5 (7.2)	NS
Social inibition	12.6 (6.4)	11.9 (5.6)	NS
Hopkins Symptom Checklist			
SCL – total score	0.86 (0.60)	0.68 (0.53)	NS (0.087)
SCL – obsessive	1.03 (0.77)	0.80 (0.69)	NS (0.085)
SCL – depression	0.87 (0.73)	0.69 (0.62)	NS
SCL – anxiety	0.91 (0.68)	0.76 (0.59)	NS
SCL – hostility	0.63 (0.69)	0.43 (0.44)	NS (0.070)
Life events (total number)	1.1 (1.0)	1.1 (1.3)	NS
Impact of events scale*	(n = 66)	(n = 40)	
IES - Total score	34.0 (16.2)	34.0 (15.4)	NS
IES - Intrusive symptoms	14.6 (7.6)	13.0 (7.0)	NS
IES - Hyperarousal	9.9 (5.7)	8.8 (5.6)	NS
IES - Avoidance	9.6 (5.8)	12.3 (5.1)	0.017

* The Impact of Event Scale was completed only by those subjects who reported at least one stressful life event in the preceeding six months.

Personality profile was similar in uncontrolled and controlled hypertensives, except for higher IES avoidance symptoms, and trends towards lower H-SCL obsessive-compulsion and H-SCL hostility in uncontrolled hypertensive patients. We, then, performed a series of logistic regression analyses to identify the predictors of blood pressure control. In a first model, we included only medical and demographic variables (Table 3, model 1) and found that age, presence of diabetes and systolic blood pressure at the first visit were the variables that best fit the model. In a second step, we included the psychological variables (type D personality, H-SCL obsessionality, and H-SCL hostility). Type D personality and H-SCL hostility did not show a significant effect and were removed from the model. On the contrary, H-SCL obsessionality improved the model (Table 3, model 2). Since IES scores were available only in the subsample of subjects who reported stressful life events (n=106), we included this score only in a third analysis. According to this model, IES avoidance symptoms were predictors of uncontrolled hypertension. The inclusion in the model of the prescription of antidepressants or benzodiazepines did not show any significant effect.

**Table 3:** Predictors of resistant hypertension (logistic regression analysis*).

	Wald (d.f. = 1)	p	OR (95% C.I.)
Model 1			
Age	12.88	0.000	1.1 (1.0-1.1)
Systolic BP	9.11	0.003	1.0 (1.0-1.1)
Diabetes	5.45	0.020	2.9 (1.2-6.9)
Model 2			
Age	7.32	0.007	1.1 (1.0-1.1)
Systolic BP	4.48	0.034	1.0 (1.0-1.1)
Diabetes	5.09	0.024	3.9 (1.2-12.8)
H-SCL obsessive	3.78	0.052	0.6 (0.3-1.0)
Model 3			
Age	3.89	0.049	1.0 (1.0-1.1)
Systolic BP	2.73	0.099	1.0 (1.0-1.1)
Diabetes	6.39	0.011	5.5 (1.5-20.3)
H-SCL obsessive	3.76	0.053	0.5 (0.3-1.0)
IES avoidance	4.59	0.032	1.1 (1.0-1.2)

* All models were adjusted for gender; Model 1: χ2 = 40.25; d.f.= 4; p < 0.001; Model 2: χ2 = 25.99; d.f.= 5; p < 0.001; Model 3: χ2 = 26.51; d.f.= 6; p < 0.001; BP: blood pressure; H-SCL: Hopkins symptom check list; IES: impact event scale.

## Discussion

The present study shows that uncontrolled hypertension is associated with low obsessionality and a large number of post-traumatic avoidance symptoms. The association remained significant after controlling for the other well known risk factors of uncontrolled hypertension, such as old age, diabetes, secondary hypertension, and isolated systolic hypertension. On the contrary, type D personality, an independent predictor of cardiovascular morbidity and mortality in patients with cardiovascular diseases, does not play any role in uncontrolled hypertension.

Anger, hostility, alexithymia, anxiety, negative affectivity and depression have been associated with hypertension [5,6]. Moreover, hypertension was frequent in patients with post-traumatic stress disorder [21], and hostility, anger, anxiety and depression were predictors of hypertension development in normotensive subjects [22]. A few studies showed also obsessive-compulsive symptoms in hypertensives [23,24,25,26]. The psychological factors associated so far with resistant or uncontrolled hypertension were anxiety and inability to channel anger [7,8]. We are not aware of any report of obsessive-compulsive symptoms in uncontrolled hypertension, even if obsessive-compulsive personality disorders have been associated with high risk of stroke, the most common complication of hypertension [27].

The combination of low obsessionality and post-traumatic avoidance symptoms among uncontrolled hypertensives suggests poor compliance to treatment. In fact, an old study showed that non compliant hypertensives had a lower average score of obsessionality than compliant patients [26]. It is noteworthy that also the other psychological predictor found in the regression analysis, i.e. avoidance symptoms, has been associated to low level of compliance among survivors to myocardial infarction [28]. The presence of avoidance symptoms after a severe stressful life event could facilitate non adherence to treatment for more than one reason. It could indicate an “avoidant coping style” that was associated with poor compliance [29]. In addition, stressor-specific avoidance, like the one measured by the IES, can be associated with some form of “denial” of the illness that lead to non adherence [30]. 

Poor adherence to medication regimes is one of most important factor influencing poor blood pressure control [31]. Different factors influence compliance and they are related to physicians, to patients and to disease [32]. The patient’s factors that make evaluation of compliance difficult are awareness and comprehension of disease, denial, and social support. It has been shown that psychological factors, such as negative emotions, sense of coherence, hostility, are important determinants on medical adherence [33]. Altogether, these data explain why conventional patient education programs have little effect on compliance of patients with hypertension [31]. In our study we did not consider the physician’s factors related to uncontrolled hypertension or compliance (e.g. clinical inertia, low patient education, complexity of prescriptions, lack of explanation, and lack of empathy) as all the patients were treated by the same medical unit according to accepted international guidelines [14].

The compliance was assessed by direct questioning by the physicians during each visit [15] and, according to patient’s responses, it was estimated to be greater than 80% in each patient. Direct questioning was deemed satisfactory to assess adherence to treatment by the physicians [15,34,35,36]. With this method we found no difference of compliance rate between uncontrolled and controlled hypertensives. In addition, no association was found between compliance and both obsessive-compulsive symptoms and IES avoidance symptoms (data not shown). It is possible that direct questioning is not applicable in uncontrolled patients, because their psychological profile affects the self assessment of compliance by direct questioning. Other methods to assess compliance are, therefore, recommended to distinguish resistant from “pseudoresistant”, uncontrolled hypertensives. They should include either determination of serum and urinary drug concentration and pill count, or a psychological approach.

To improve compliance it may be necessary to increase patient motivation and understanding of the disease and of the antihypertensive treatment but also to identify psychological characteristics of those patients who probably don’t remember unconsciously to take their medications or psychological factors that could bring to a distorted perceived severity of illness. This approach is probably more cost effective than an extensive work up for secondary hypertension in uncontrolled patients, as the psychological profile was more strongly associated with uncontrolled hypertension than the type of hypertension (*i.e.*, primary vs secondary).

Other hypotheses to be taken into consideration to explain the link between low obsessionality and post-traumatic avoidance symptoms and uncontrolled hypertension include the effects of hypertensive drugs on psychological profile. For instance, clonidine is particularly beneficial in patients with obsessive-compulsive disorders, probably because of its antiadrenergic effects [37]. However, only four subjects of our study cohort were taking this drug, and we are not aware of any other study showing any influence of antihypertensive drugs on the psychological profile.

Uncontrolled hypertensives are at high risk for cardiovascular events due to poor blood pressure control, which is a well known morbidity and mortality risk factor [4,38]. In our study, type D personality was not associated with resistant hypertension or with previous cardiovascular events, while it was associated with increased morbidity in patients with cardiovascular diseases [12]. The cross-sectional design of our study does not allow speculations on the impact of type D personality on cardiovascular risk in patients with uncontrolled hypertension. However, type D personality was associated with high level of depression, anxiety, and hostility [13] and negative affectivity was associated with obsessive-compulsive disorder [39]. The present study extends those observations and shows, for the first time, that type D personality encompasses also compulsive symptoms. Type D personality may have, therefore, opposite effects on blood pressure control and cardiovascular risk. Some psychological characteristics of type D personality may play a role in the pathogenesis of the cardiovascular events and death (depression, anxiety and hostility), but others may be protective towards hypertension through better compliance and blood pressure control (compulsive symptoms) [40].

The present study has some limitations that should be taken into consideration: we used self-reported questionnaires instead of diagnostic interviews, and some patients refused to participate. However, even with these limitations, we were able to confirm the reported association between type D personality and malignancy [41], which supports the adequacy of the sample and of the study protocol.

## Experimental Section

All the consecutive patients assessed at the Hypertension Outpatient Clinic of the University of Padua Medical School between October 1st and December 31st, 2005 (n = 266) were invited to participate to the study, which had been approved by the local Ethical Committee, and 205 (38 coming for the first time) gave an informed written consent to use their data in a confidential form. All patients received follow up visits until October 2006. The study design was explorative, observational and longitudinal.

Screening for secondary hypertension (with dosage of plasma creatinin, potassium, plasma renin activity and aldosterone, urinary catecholamine, plasma cortisol, thyroid ormone, renal duplex doppler sonography and renal scan) and tests to define the cardiovascular risk were performed at the first visit. The patients were classified as “essential” or “secondary”, according to the results of the diagnostic work up, which had to be completed by the end of the study. In each visit blood pressure was measured three times in the sitting position with a mercury sphygmomanometer, with a properly-sized cuff and after the patient had been sitting quietly for five minutes. The mean of the three readings was used for the analysis of the results.

Antihypertensive drugs were prescribed according to the 2003 European Society of Hypertension / European Society of Cardiology guidelines [14]. Compliance was assessed by direct questioning [15]. At follow up, they were classified as “controlled”, if clinic sitting blood pressure was below 140/90 mmHg (130/80 in the presence of diabetes or nephropathy), or “uncontrolled”, if it was above the same limits, despite reported adherence to treatment with at least three antihypertensive medications. Patients taking four or more medications, including a diuretic, were defined as “uncontrolled” [3]. “White coat effect” was excluded with 24 hour ambulatory blood pressure monitoring in all the hypertensives with office uncontrolled hypertension but who referred good home blood pressure control.

The following data were collected for each patient: Blood pressure at first and last visit, number of outpatient consultations, body mass index (BMI), duration of hypertension, type and number of antihypertensive drugs, chronic use of benzodiazepine or antidepressant, presence of diabetes, previous cardiovascular or cerebrovascular events, and history of malignancy.

A series of self-reported questionnaires to investigate the psychological factors was self administered at the time of the outpatient visit. Type D personality was assessed with the 16-item DS16 which was developed and validated in Belgian cardiac patients [16] and in other samples of various nationalities [17,18]. The questionnaire was translated in Italian using the back translation method. Principal component analysis (PCA) and Cronbach’s alpha have been used to study the psychometric properties of the Italian version of the questionnaire. PCA demonstrated the presence of two main factors that overlap those of the original version (data not shown), and the Cronbach’s alpha showed a good reliability of the questionnaire (α = 0.85 for the ‘negative affectivity’ subscale, and α = 0.73 for the ‘Social Inhibition’ subscale). Patients were classified as having a ‘type D Personality’ if they scored above the median for both subscales (11 in both subscales), as described in previous papers [16,17,18].

The other self-reported questionnaires included: a) the short version of the Hopkins Symptoms Check List (H-SCL) [19, Cronbach’s α=0.93] to measure general psychopathology; b) a life event questionnaire, an inventory developed for this study that investigates the occurrence of stressful life events in the previous 6 months; c) the revised version of the Impact of Events Scale (IES), designed to assess current subjective distress for any specific life event [20, Cronbach’s α = 0.89] for those subjects who reported a stressful life event (n = 106). To assess life events, we used a modified version of the Life Experiences Survey, consisting of 14 items that investigate the occurrence of: serious illness, accident, physical aggression, serious illness or physical aggression of a loved one, loss of a loved one, separation or divorce, interpersonal conflicts, loss of work, economical problems, problems with law [42]. Patients were asked to indicate which events had occurred during the six months prior to the administration of the questionnaire and at the end of the questionnaire they could name other events not mentioned in the list. 

### Statistical analysis

Pearson’s chi-squared test and Student’s t-test were used to compare groups. A logistic regression analysis was used to derive a multivariate model of the risk of uncontrolled hypertension, while taking into account potential confounder variables. The results are given as mean ± SD. The significant level was set at p < 0.05. 

## Conclusions

In conclusion, the present study, showing unforeseen psychological factors in uncontrolled hypertension, strongly supports a multidimensional approach to the patients with hypertension to focus not only the cardiovascular risk factors, but also the psychological characteristics that enhance the cardiovascular risk through emotional stress and poor compliance to treatment.
